# Brain-Dead Patients are not Cadavers: The Need to Revise the Definition of Death in Muslim Communities

**DOI:** 10.1007/s10730-012-9196-7

**Published:** 2012-09-28

**Authors:** Mohamed Y. Rady, Joseph L. Verheijde

**Affiliations:** 1 Department of Critical Care, Mayo Clinic Hospital, 5777 East Mayo Boulevard, Phoenix, AZ 85054 USA; 2 Department of Physical Medicine & Rehabilitation, Mayo Clinic, 13400 E Shea Blvd, Scottsdale, AZ 85259 USA

**Keywords:** Brain death, Cardiac death, End-of-life care, Intensive care, Islam, Legal death, Organ donation, Muslim communities

## Abstract

The utilitarian construct of two alternative criteria of human death increases the supply of transplantable organs at the end of life. Neither the neurological criterion (heart-beating donation) nor the circulatory criterion (non-heart-beating donation) is grounded in scientific evidence but based on philosophical reasoning. A utilitarian death definition can have unintended consequences for dying Muslim patients: (1) the expedited process of determining death for retrieval of transplantable organs can lead to diagnostic errors, (2) the equivalence of brain death with human death may be incorrect, and (3) end-of-life religious values and traditional rituals may be sacrificed. Therefore, it is imperative to reevaluate the two different types and criteria of death introduced by the Resolution (Fatwa) of the Council of Islamic Jurisprudence on Resuscitation Apparatus in 1986. Although we recognize that this Fatwa was based on best scientific evidence available at that time, more recent evidence shows that it rests on outdated knowledge and understanding of the phenomenon of human death. We recommend redefining death in Islam to reaffirm the singularity of this biological phenomenon as revealed in the Quran 14 centuries ago.

## Background

Human death is categorized into two alternative types (neurological or cardiorespiratory) in organ donation (The President’s Council on Bioethics 2008). The neurological criterion of death is the irreversible loss of consciousness and spontaneous respiration in heart-beating organ donors (Wijdicks et al. 2010). The circulatory criterion of cardiorespiratory death is 2–5 minutes of absent arterial pulse in non-heart-beating donors (Bernat et al. 2010). There is no medical or scientific evidence validating either the neurological or circulatory criterion of death being synonymous with true biological death (Bellomo and Zamperetti 2007; Rady et al. 2010; Tibballs 2008; Zamperetti et al. 2004). Therefore, the binary categorization of human death fails to accurately characterize the singularity of this natural biological phenomenon. Lacking this scientific validation, defining death in organ donation essentially follows a social construct model, one that can only be justified by reinterpreting human death philosophically and considering dying patients as human cadavers (Bernat 2010; Iltis and Cherry 2010; Khushf 2010; Shewmon 2010). Western scholars have put forth several philosophical rationales to justify both the neurological and the circulatory criteria of death. In regard to the neurological criterion of death, they proposed loss of capacity for consciousness, loss of personhood, loss of essence of being human, and loss of ability to interact with external environment (Collins 2010; Khushf 2010; Lee and Grisez 2012; The President’s Council on Bioethics 2008). Critics have identified serious weaknesses in the philosophical justifications of this criterion of death (Joffe 2010; Nair-Collins 2010; Shewmon 2001, 2009; Thomas 2012; Zamperetti and Bellomo 2009). The circulatory criterion of death has been justified philosophically by arguing that there is no intent or action to attempt resuscitation in a patient consenting to organ donation when the spontaneous heartbeat (arterial pulse) ceases, even though the heart can be restarted with recovery of brain functions (Bernat 2006, 2010). Different commentators have pointed out flaws in the philosophical justification of this criterion of death as well (Bellomo and Zamperetti 2007; Iltis and Cherry 2010; Joffe et al. 2011; Marquis 2010; Rady et al. 2010; Veatch 2010; Zamperetti et al. 2009).

In this article, we summarize contemporary scientific evidence proving that the neurological and circulatory criteria of death in organ donation are noncompliant with the definition of death in Islam. The Resolution (Fatwa) of the Council of Islamic Jurisprudence on Resuscitation Apparatus (1986) introduced two different types and criteria of human death in Islam (Albar 1996). We outline three ways how adopting a faulty criterion of death in organ donation has unintended consequences for dying Muslim patients: (1) the expedited process of determining death by neurological or circulatory criterion for the purpose of rapid retrieval of transplantable organs may lead to diagnostic errors, (2) the widely assumed equivalence of brain death with human death may be proven incorrect, and (3) religious values and traditional rituals may be sacrificed in end-of-life care. We conclude that: (1) alternative solutions must be considered and prioritized to decrease transplantation demand in society, and (2) the two alternative types and criteria of death in Islam should be reevaluated to reaffirm the singularity of this biological phenomenon as revealed in the Quran 14 centuries ago.

## History of the Western Definition of Death

Brain death, as synonymous with human death, has been deeply rooted in Western medical practice as a prerequisite for advancing human organ transplantation over the past 40 years (Rady and Verheijde 2011). Binary categorization of death has fulfilled the utilitarian goal of increasing the supply of organs at the end of life. Here, we use the term “utilitarian” to mean a philosophical theory “designed [customized] for or capable of a particular function or use often to the exclusion of values” (The Free Dictionary by Farlex 2012). This particular understanding of utilitarianism was the subject of Pope John Paul’s II criticism stating: “[i]n our own day, history is in a way repeating itself. Utilitarianism is a civilization of production and of use, a civilization of “things” and not of “persons”, a civilization in which persons are used in the same way as things are used” (Pope John Paul II 1994).

The publication of the *Report of the Ad Hoc Committee of the Harvard Medical School to Examine the Definition of Brain Death* marked the formal introduction of the new definition of death in Western medicine (Beecher and Ad Hoc Committee of the Harvard Medical School to Examine the Definition of Brain-death 1968). Although Wijdicks (2003) concluded that organ transplantation was not the primary driver of conceptualizing the neurological criterion of death, his analysis of historical documents about the forming of the Ad Hoc Committee and the drafting of the final report did not support this conclusion. Wijdicks (2003) described the substantial contribution of the surgical transplantation practice in composing the membership of the Ad Hoc Committee and facilitating the expedited approval and endorsement of this “new definition of death”. Henry Beecher, MD (chair of the committee) wrote in a letter to Joseph Murray, MD (transplant surgeon on the committee):

I cannot tell you how strongly I agree with you that it would be most desirable for a group at Harvard University to come to some subtle conclusion as to a new definition of death. Murray responded …“Can society afford to lose organs that are now being buried?” is the most important one of all. Patients are stacked up in every hospital in Boston and all over the world waiting for suitable donor kidneys. At the same time patients are being brought in dead to emergency wards and potentially useful kidneys are being discarded. (Wijdicks, 2003)

Wijdicks (2003) described the attitude of transplant surgeons at the time when the Ad Hoc Committee was being formed:

Murray recalls… “it seemed everyone wanted a piece of the action and the attendees—mostly young, aggressive, and ambitious doctors—jockeyed for prominent positions in the field” (Murray, 2001)….[C]ertain doctors at the kidney conference who had stood up and said, “I am not going to wait for the medical examiner to declare the patient dead. I’m just going to take the organ.” Boston newspaper press decreed that the “Brigham doctors were playing god by removing organs”. (Murray 2001)

Wijdicks (2003) also noted the strong desire of Murray to replace the term *irreversible coma* with *death* in the final report:

He [Murray] did not agree with the term irreversible coma, but his edit to replace it with “death” did not make the final draft …. The term irreversible coma now seems even more confusing and Murray suggests leaving it out altogether.

The contribution of ethicists, theologians, and the general public in the Committee’s deliberation and final report was kept at a minimum to avoid dissenting opinions. This facilitated an expeditious approval and publication of the final report:

There was a perceived need with most members to complete the work quickly…. Transplantation was not actually mentioned, but correspondence suggests that there was appropriate sensitivity not to link this work to transplantation

…Potter (one of the youngest members and later founding fellow of the Hastings Center) was startled by the urge to finish the document. He felt some issues were unsettled and more discussions were needed…. (Wijdicks 2003)

It is claimed that advances in intensive care and artificial life support had been the main driver of characterizing irreversible coma as human death and enabling the withdrawal of artificial life support (Khushf 2010; Wijdicks 2003). This claim can be dismissed because withdrawing nonbeneficial life-support treatment and providing medically appropriate end-of-life care is permissible in dying patients without the need of equating irreversible coma with human death. However, equating irreversible coma with human death is a prerequisite in procuring transplantable organs legally from these patients without contravening homicide laws.

## Definition of Death in Muslim Communities

The Western definition of death has been introduced in Muslim communities to promote organ donation and transplantation (Al-Mousawi et al. 1997; Choo 1995; Haque 2008; Moosa 1993). The Resolution (Fatwa) of the Council of Islamic Jurisprudence on Resuscitation Apparatus (1986) incorporated the concept of brain death into the legal definition of death in Islam:

[A] person is pronounced legally dead and consequently, all dispositions of the Islamic law in case of death apply if one of the two following conditions has been established: (1) there is total cessation of cardiac and respiratory functions, and doctors have ruled that such cessation is irreversible; (2) there is total cessation of all cerebral functions and experienced specialized doctors have ruled that such cessation is irreversible and the brain has started to disintegrate. (Albar 1996)

This juridical resolution (fatwa) marked the formal introduction of two different types and criteria of human death in Islam. Muslim countries adopted this definition of death after the enactment of the Uniform Determination of Death Act (UDDA) in the United States, which stated that:

An individual who has sustained either (1) irreversible cessation of circulatory and respiratory functions, or (2) irreversible cessation of all functions of the entire brain, including the brain stem, is dead. A determination of death must be made in accordance with accepted medical standards. (National Conference of Commissioners on Uniform State Laws 1981)

The expansion of organ donation and transplantation in Muslim communities is dependent upon accepting that heart-beating donors are deceased and thus should be considered human cadavers (Raza and Hedayat 2004; Zurani et al. 2010). The practice of what has been called *cadaveric* organ procurement appears to have expanded rapidly in these communities (Mandegar et al. 2009; Masri et al. 2004; Najafizadeh et al. 2009; Saudi Center for Organ Transplantation 2010). For instance, in a review of end-stage chronic kidney disease in Saudi Arabia, Al-Sayyari and Shaheen (2011) argued there was a need to boost organ donation and procurement rates. The authors concluded “[a]lthough this is likely to face societal resistance, we need to explore the introduction of non-heart-beating donation, and the presumed consent strategies” (Al-Sayyari and Shaheen 2011). This strategy was intended to increase the organ supply and meet the future demand for transplantable organs because of a rapidly rising incidence of end-stage kidney disease in Saudi Arabia. In a separate study of brain-dead patients admitted to the intensive care unit at the same hospital, Alsultan (2011) reported those who had surgical procurement in accordance with the heart-beating organ-donation protocol had shorter lengths of stay in the intensive care unit than did patients who died naturally after a diagnosis of brain death was made. Alsultan did not conclude that patients declared brain dead had died prematurely, but instead, he reasoned that early death secondary to heart-beating organ donation was useful because of “…a better utilization of the intensive care units’ beds by other than brain-dead patients would not produce great cost savings, but may provide care for more patients with better quality of care” (Alsultan 2011). In both articles (Al-Sayyari and Shaheen 2011; Alsultan 2011), utilitarian reasons were used to justify the introduction of non-heart-beating donation and the expansion of heart-beating donation. In the first article, Al-Sayyari and Shaheen (2011) forecasted a rising demand for kidney transplantation that required a more pragmatic approach to defining death. In the second article, Alsultan (2011) advocated better utilization of intensive care resources at the cost of patients declared brain dead so as to create more opportunities to care for patients with diagnoses other than devastating neurological injuries. In a separate study of brain-dead patients admitted to intensive care units, Najafizadeh et al. (2012) reported on developing specialized intensive care units at transplant centers that were designated for receiving and resuscitating potential brain-dead donors before surgical procurement. Potential donors were transferred from surrounding affiliated hospitals and distant cities to the designated organ procurement units at transplant centers who specialized in preserving transplantable organs in potential brain-dead donors before surgical procurement (Najafizadeh et al. 2012). The above strategies would increase the yield of transplantable organs (e.g., hearts) in Muslim communities (Canver et al. 2011; Kazemeyni et al. 2009; Mandegar et al. 2009; Najafizadeh et al. 2012). The availability of heart-beating donors would also increase so that “[a] hospital in a Muslim country can increase cardiac transplant activity….comparable to that in worldwide counterparts” (Canver et al. 2011). Utilitarian reasoning would be invoked to facilitate end-of-life organ procurement because “…it is a pity to waste such candidate cadavers without trying to save the life of many others who need their organs” (Albar 2012). The advocated policies, in transplantation practice, projected a future trend of willingness to increase the supply of transplantable organs and perhaps to sacrifice traditional Islamic end-of-life care of the dying in Muslim communities.

## Determination of Brain Death

In an independent analysis, the President’s Council on Bioethics (2008) concluded that there was insufficient clinicopathological evidence to validate brain death determination and its equivalence to biological death (Table 1).

**Table 1:** Physiological evidence of somatic integration in brain-dead individuals

Homeostasis of a countless variety of mutually interacting chemicals, macromolecules and physiological parameters, through the functions especially of liver, kidneys, cardiovascular and endocrine systems, but also of other organs and tissues (e.g., intestines, bone and skin in calcium metabolism; cardiac atrial natriuretic factor affecting the renal secretion of renin, which regulates blood pressure by acting on vascular smooth muscle; etc.) Elimination, detoxification and recycling of cellular wastes throughout the body Energy balance, involving interactions among liver, endocrine systems, muscle and fat Maintenance of body temperature (albeit at a lower than normal level and with the help of blankets) Wound healing, capacity for which is diffuse throughout the body and which involves organism-level, teleological interaction among blood cells, capillary endothelium, soft tissues, bone marrow, vasoactive peptides, clotting and clot lysing factors (maintained by the liver, vascular endothelium and circulating leucocytes in a delicate balance of synthesis and degradation), etc. Fighting of infections and foreign bodies through interactions among the immune system, lymphatics, bone marrow, and microvasculature Development of a febrile response to infection Cardiovascular and hormonal stress responses to unanesthetized incision for organ retrieval Successful gestation of a fetus in a [brain dead] pregnant woman Sexual maturation of a [brain dead] child Proportional growth of a [brain dead] child	

Table is reproduced from The White Paper by The President’s Council on Bioethics on “Controversies in the determination of death” (The President’s Council on Bioethics 2008) and source (Shewmon 2001). Materials produced by the President’s Council on Bioethics are government documents and in the public domain. Please note the source as http://bioethics.georgetown.edu/pcbe/

Although the American Academy of Neurology guideline is the most commonly used method for determining brain death worldwide, the 2010 update of the evidence-based guideline has assigned only the level “U” of strength of scientific evidence (inadequate or conflicting data and predictor or test unproven) to several elements included in the process of brain death determination (Wijdicks et al. 2010). In a US survey, Joffe et al. (2012) reported that the majority of neurologists lacked a clear understanding of the diagnostic accuracy of tests that were performed in determining brain death and the rationale of equating this neurological diagnosis with human death. The limitations of the American Academy of Neurology guideline can be summarized as follows:

1. clinical bedside determination of the absence of inner and external awareness can be erroneous because of motor paralysis (Joffe et al. 2010a; Karakatsanis 2008),
2. certain neurological functions are retained in brain-dead patients (Joffe 2010; Rady et al. 2010; Rasulo et al. 2010),
3. some neurological reflexes are reversibly lost and recover with time (Roberts et al. 2010; Webb and Samuels 2011),
4. the histopathological examination of brains in 60 % of donors determined to be brain dead have normal or minimal ischemic injury of the brainstem (Wijdicks and Pfeifer 2008),
5. intracranial blood flow and circulation continue in some brain-dead donors (Wijdicks 2010),
6. the American Academy of Neurology guideline rejects that whole brain disintegration or necrosis is an essential requirement to verify brain death in donors before heart-beating surgical procurement (Wijdicks and Pfeifer 2008),
7. brain-dead patients continue to have coordinated biological, homeostatic and cardiovascular functions (Shewmon 1998, 2001, 2009; The President’s Council on Bioethics 2008),
8. the perceived pressure to expedite, rush through brain-death determination, and procure transplantable organs (Lustbader et al. 2011; Varelas et al. 2011) can lead to diagnostic errors and mistakenly identifying recoverable conditions as unrecoverable (Marik and Varon 2010; Morales 2008; Ornstein and Weber 2007; Powner 2009; Sullivan et al. 2012), and,
9. the apnea test, requiring elevation of arterial carbon dioxide, does not ascertain the irreversible cessation of brainstem respiratory function and the test itself can precipitate transtentorial herniation and fatal outcome in patients whose conditions are potentially recoverable (Joffe et al. 2010b; Shewmon 2012).

## Determination of Cardiorespiratory Death

The circulatory criterion of determining cardiorespiratory death in non-heart-beating organ donation is 2–5 minutes of absent arterial pulse (Bernat et al. 2010). However, there are scientific flaws with this criterion: (1) the heart is capable of recovering its mechanical function and spontaneous regular beating, and (2) the whole brain, including the brainstem, can remain viable after 2–5 minutes of absent arterial pulse (Joffe et al. 2011; Rady et al. 2010). Therefore, the ceased physiological functions of the cardiovascular, respiratory, and neurological systems are reversible at the time of procuring organs from non-heart-beating donors. Clinical evidence disproving the circulatory criterion of cardiorespiratory death includes: (1) the unassisted recovery of spontaneous circulation (also called Lazarus phenomenon) is possible after 10–15 minutes of circulatory arrest, (2) the structural integrity and functional recovery of the central nervous system can be preserved after prolonged periods of circulatory arrest, (3) the hearts procured after circulatory arrest regain normal function in transplant recipients, and (4) the artificial circulation that is initiated for preserving organs after circulatory arrest can resuscitate vital signs of donors during surgical procurement (Bellomo and Zamperetti 2007; Joffe et al. 2011; Rady et al. 2010; Zamperetti et al. 2003, 2009).

## Medicolegal Definition of Death in Muslim Communities

The 2010 update of the American Academy of Neurology guideline for determining brain death fails to meet the three essential requirements stated in the Islamic definition of death: (1) total cessation of all brain functions, (2) irreversibility of cessation, and (3) the onset of disintegration of the brain (Rady et al. 2009). The failure to meet the standard definition of death is exemplified by the fact that some brain-dead patients retain: (1) electrical activity of the cerebral cortices (on electroencephalogram), (2) viable neural pathways between the cerebral hemispheres and brainstem, and (3) normal histopathology as shown by the examination of brain structures on autopsy (Joffe 2010; Nair-Collins 2010; Rady et al. 2010). The circulatory criterion of determining cardiorespiratory death in non-heart-beating donation does not fulfill the requirement of irreversible cessation of cardiorespiratory functions (Joffe et al. 2011; Potts et al. 2010; Veatch 2010). Both the heart and the brain are capable of recovering respective functions in non-heart-beating donors (Joffe et al. 2011; Marquis 2010). Therefore, in both heart-beating (Canver et al. 2011) and non-heart-beating (Faraj et al. 2010) donors, organs are likely to be surgically procured from patients who do not fulfill the Islamic definition of death approved in 1986. For the same reasons, Western scholars have criticized the noncompliance of organ donation from heart-beating (Nair-Collins 2010) and non-heart-beating (Harrington 2009) donors with the stipulated accepted medical standard of determining death in the UDDA.

Organ donation has been permitted in Muslim communities based on the premise that the process upholds certain Islamic principles: (1) the preservation of dignity of the living and deceased human bodies; (2) the avoidance of harm to donors; (3) the duty of seeking cure from diseases; and (4) the ownership of the human body by God, the Creator (Albar 1996). These Islamic principles are difficult to reconcile with the contemporary medical understanding of organ donation and transplantation: (1) preserving and procuring organs entails performing invasive surgical procedures on living and deceased donors; (2) removing vital organs can potentially end the donor’s life; (3) transplanting an organ is not a permanent cure of organ failure in the recipient because the immune system inevitably rejects the donor’s organ; and (4) donating and receiving organs between individuals contravenes the divine ownership of the human body. In regard to the latter, God endowed man with a complex immune system that recognizes foreign tissues and organs. The immune system has evolved to ultimately reject transplanted organs because they are not native of that particular human body (Nankivell and Kuypers 2011).

There are two consequences of using a medically faulty criterion of death in organ donation. First, organ procurement is performed in the operating room with no general anaesthesia because donors are presumed dead (Fitzgerald et al. 2003; Rodriguez-Arias et al. 2011; Young and Matta 2000). Movements in heart-beating donors are commonly attributed to spinal reflexes (Saposnik et al. 2005). However, normal or minimally injured brainstem neural pathways are found at autopsy in over 60 % of heart-beating donors (Wijdicks and Pfeifer 2008). This suggests that the neurological integration may be present at a higher level than the spinal cord. Neuromuscular-blocking agents may be given to induce paralysis without administering general anaesthesia during surgical procurement (Young and Matta 2000). Nociception and awareness in donors cannot be excluded during this surgical procedure (Fitzgerald et al. 2003; Rady et al. 2010; Verheijde and Rady 2011). Similarly, the viability and function of the central nervous system may be preserved in donors declared dead by the circulatory criterion of cardiorespiratory death. Continuous electroencephalographic recordings in brain-injured non-heart-beating donors have detected the presence of electric activity in higher brain structures (Auyong et al. 2010). Higher brain activity from nociception and awareness cannot be ruled out in some donors (Rady and Verheijde 2010). Critics have commented on the potential harm of surgically procuring organs without administering appropriate general anaesthesia to non-heart-beating donors who can retain residual brain functions (Cochrane and Bianchi 2011; Glannon 2011; Rodriguez-Arias et al. 2011).

Second, donors are not legally dead if they do not fulfill the criterion of death stipulated in the Resolution of the Council of Islamic Jurisprudence on Resuscitation Apparatus (1986) (Albar 1996). Therefore, the surgical procurement then necessarily becomes the proximate cause of death. Muslim scholars consider the rule of “presumption of continuity” when determining the consequences of performing specific actions in the presence of a reasonable doubt (Badawi 2011). For example “…if it is uncertain if a patient is dead (however evolving definition of death is accepted), then continuity of life should be presumed until death otherwise is confirmed” (Badawi 2011). Therefore, the Islamic rule of “presumption of continuity” is rejected if organs are procured from donors who are determined dead by an ambiguous or flawed criterion of death.

## Islam and End-of-Life Organ Donation

The clinical diagnosis of brain death is consistent with a diagnosis of a catastrophic neurological condition (i.e., irreversible coma) and is similar to other neurological conditions within the spectrum of states of impaired consciousness in human beings (Rady et al. 2009; Verheijde et al. 2009; Zamperetti et al. 2004). From a theological perspective in Islam, the neurological condition of brain death does not represent complete death (Bedir and Aksoy 2011; Padela et al. 2011b; Sachedina 2000). Padela et al. (2011b) have pointed out the serious gaps in contemporary medical understanding and clinical diagnosis of brain death and its endorsement as human death in the Islamic faith. These gaps pertained to: (1) the retention of residual brain functions; (2) the recovery of some previously ceased brain functions; (3) the absence of whole brain degeneration and necrosis; and (4) the uncertainty of medical tests and bedside examination in determining this condition with reasonable accuracy (Padela et al. 2011a). Bedir and Aksoy have concluded brain-dead patients should be cared for as living humans who could still suffer from surgical procedures:

…it can be asserted that brain death is not absolute death according to Islamic sources; for in the patients diagnosed with brain death the soul still has not abandoned the body. Therefore, these patients suffer in every operation performed on them. (Bedir and Aksoy 2011)

Professor Sachedina has acknowledged that the Western concept of death that equated brain death with human death was incompatible with Islamic teachings because:

….the Qur’anic view of human person, the nafs [soul], that rejects the dichotomization of human personality into a body and mind, is at the root of theological debate on the relationship between life and death. As a nafs who dies through the divine decree any definition of this nafs’s death must focus on the criteria that determines the death of the whole human rather than just a part of his biological existence. In other words, no definition of death that fails to take a living person, as seen in the Qur’an, can have a valid ground for acceptance in Islamic jurisprudence. (Sachedina 2000)

Scholars in other Abrahamic faiths (Judaism and Christianity) have argued that the theological concept of death and departure of the soul from the human body was not necessarily consistent with the neurological diagnosis of brain death (Kunin 2004; Verheijde and Potts 2010). The debate on the definition of death for donating organs in Judaism has been reintensified as some Orthodox Jewish scholars have rejected the neurological criterion of death (The Lancet 2011). There is also a growing opinion within Christianity that the secular definition of death for organ donation is not the same as the theological definition of human death (Tonti-Filippini 2012).

Multiple surveys have suggested that religious values and beliefs have influenced the sociocultural attitudes toward organ donation in Muslim communities (Alghanim 2010; AlKhawari et al. 2005; Aslam and Hameed 2008; Sharif et al. 2011; Yilmaz 2011). Khalid et al. (2012) have reported that most healthcare professionals and families were unwilling to accept the concept of organ donation from brain-dead patients. Transplant advocates have made several arguments to overcome the religious barriers toward organ donation:

…We need to acknowledge that most *religious scriptures were written hundreds, if not thousands of years ago, before any consideration of organ transplantation*. Consequently, any religious position on organ donation is subject to a religious scholar’s interpretation of the scriptures *and the values espoused by the faith*. [Emphasis added] (Randhawa 2012);

…organ transplantation is a relatively new medical procedure, there is *no explicit reference to it in many original religious texts* [Emphasis added] (Randhawa et al. 2010);

…[a] *dying person may not need his viable organs* as much as persons on waiting lists, where an organ transplant could make a difference [Emphasis added] (Khalid and Khalil 2011); and

…Scholars have debated the contemporary issue of organ donation and transplantation based on their interpretation of Qu’ranic verses. *Although violation of the human body, whether alive or dead, is forbidden in Islam a greater emphasis is placed on altruism and humanitarian need*. “If anyone saved a life, it would be as if he saved the life of the whole people” [(The Holy Quran)] is a shared principle among the Abrahamic religions *and provides theologic justification for enacting the Islamic juristic principle of al*-*darurat tubih almahzurat or “necessity overrides prohibition”.* [Emphasis added]. (Sharif et al. 2011)

The above arguments implicitly attempt to: (1) contest the authenticity and divinity of religious scriptures and teachings, (2) raise doubt about the relevancy of traditional religious values and beliefs in transplantation medicine, (3) reinterpret the moral permissibility of religiously prohibited actions in exceptional circumstances, and (4) reinterpret the divine moral code set forth in Abrahamic faiths to satisfy societal needs. The Quran and the Sunnah are considered the ultimate sources of reference in Islam whenever there are differences in interpreting the moral permissibility of an action, e.g., organ donation at the end of life (Rady et al. 2009). The Quran is the divine revelation from God to mankind and the Sunnah is the tradition of the Prophet Muhammad: what he said, what he did, what he saw, and approved during his lifetime.

First, the Quran reaffirms the authenticity and divinity of the Abrahamic faiths’ scriptures:

It is He Who has sent down the Book (the Qur’an) to you (Muhammad SAW) with truth, confirming what came before it. And he sent down the Taurat (Torah) and the Injeel (Gospel) (3) Aforetime, as a guidance to mankind, And He sent down the criterion [of judgement between right and wrong (this Quran)]. (Chapter 3: verses 2-3) (The Holy Quran 2012)

Second, the moral code and values revealed in the Quran and the Sunnah are relevant in determining the acceptability of questionable human actions including those in organ donation and transplantation:

(And) if you differ in anything amongst yourselves, refer it to Allah [God] and His Messenger (SAW), if you believe in Allah [God] and in the Last Day. That is better and more suitable for final determination. (Chapter 4: verse 59) (The Holy Quran 2012)

Third, the Quran sets the rules and limits between permissible and forbidden actions through the revelation of the Al-Ahkam (commandments), Al-Faraid (obligatory duties) and Al-Hudud (legal laws for the punishment): “…And whoever transgresses the limits ordained by Allah [God], then such are the Zalimun (wrong-doers)” (Chapter 2: verse 229) (The Holy Quran 2012). These rules determine reinterpreting the moral permissibility of forbidden actions in exceptional circumstances. The rules are intended to protect religion, life, mind, property, and progeny, in that order of priority. Opinions (fatwas) on the moral permissibility of human actions should adhere to these rules (Table 2): (1) certain actions are explicitly forbidden; (2) the divine commands extend to all areas of life and every field of action; and (3) although necessity allows prohibited matters, the prevention of evil has priority over obtaining benefit (Al-Allaf 2003; Padela 2007; Rady et al. 2009).

**Table 2:** Some of the general principles of Islam for rendering opinions on the moral status of actions and the five ruling values of actions with their consequences in the here and now and in the hereafter

Rules for opinions on moral status of actions
God alone defines the standard of right and wrong
Good deeds are good only because God commands them, and evil is evil because God forbids it
God’s commands are purposeful and, as such, His will extends to all areas of life and every field of action
Need and necessity are equivalent
Necessity allows prohibited matters
Injurious harm should be removed
Prevention of evil has priority over obtaining benefit
The greater benefit prevails over the lesser benefit
Five ruling values of actions: consequences in the here and now and the hereafter
Obligatory or required (Wajib/Fardh): God rewards for performance and punishes for neglect
Recommended (Mustahaab/Mandoob): God rewards for performance but does not punish for neglect
Permitted (Mubah): God neither rewards for performance nor punishes for neglect
Discouraged or abominable (Makrooh): God punishes for performance and rewards for avoidance
Forbidden or prohibited (Haram): God punishes for performance and rewards for avoidance

Table is reproduced from (Rady et al. 2009) and sources (Al-Allaf 2003; Padela 2007)

Transplant advocates claim that the Quran and the Sunnah do not directly refer to organ donation and transplantation (Randhawa et al. 2010; Sharif et al. 2011). Therefore, the limits between permissible and forbidden actions can be reinterpreted in favor of promoting willingness toward donating organs. There are three concerns with this approach. First, the Quran cautions against misinterpreting or seeking hidden meanings to distort the truth about the divine moral code:

It is He Who has sent down to you (Muhammad SAW) the Book (this Qur’an). In it are Verses that are entirely clear, they are the foundations of the Book [and those are the Verses of Al-Ahkam, Al-Faraid and Al-Hudud]; and others not entirely clear. So as for those in whose hearts there is a deviation (from the truth) they follow that which is not entirely clear thereof seeking Al-Fitnah, and seeking for its hidden meanings [seeking discord and seeking an interpretation suitable to them], but none knows its hidden meanings save Allah [God]. (Chapter 3: verse 7) (The Holy Quran 2012)

Second, the Quran refers to human bodily parts in several verses and emphasizes the personal entrustment and responsibility, for example:

1. the Quran warns about changing the nature of God’s creation of man:
  “… [Satan said]: and indeed I will order them to change the nature created by Allah [God]” (Chapter 4: verse 119),
  “….Allah [God] has not made for any man two hearts inside his body” (Chapter 33: verse 4) (The Holy Quran 2012);
2. the Quran describes that each person bears the responsibility of his (her) own body parts in the earthly life and the Hereafter:
  “…On the Day when their tongues, their hands, and their legs (or feet) will bear witness against them as to what they used to do” (Chapter 24: verse 24),
  “…Till, when they reach it (Hell-fire), their hearing (ears) and their eyes, and their skins will testify against them as to what they used to do” (Chapter 41: verse 20) (The Holy Quran 2012); and
3. the human body is an “amanat” entrusted by God to that person:
  “O’ you who believe! Betray not Allah [God] and His Messenger, nor betray knowingly your Amanat (things entrusted to you, and all the duties which Allah [God] has ordained for you)” (Chapter 8: verse 27) (The Holy Quran 2012).

Third, the Quran’s definition of human death is incompatible with the medical criterion of death in organ donation. The moment of death is the time when all the signs of life have ceased irreversibly and the soul has departed the body:

1. the Quran describes that the signs of life are present as long as either the brain and/or the heart are capable of functioning:
  “Then He fashioned him in due proportion, and breathed into him the soul (created by God for that person), and He gave you hearing (ears), sight (eyes) and hearts. Little is the thanks you give!” (Chapter 32: verse 9) (The Holy Quran 2012);
2. the Quran describes man’s limited knowledge of what constitutes the human soul, the locus of the soul in the body, and the moment when the soul is departing from the body permanently:
  “And they ask you (O Muhammad SAW) concerning the Ruh (the Spirit); Say: The Ruh (the Spirit): is one of the things, the knowledge of which is only with my Lord. And of knowledge, you (mankind) have been given only a little” (Chapter 17: verse 85),
  “It is Allah [God] Who takes away the souls at the time of their death, and those that die not during their sleep. He keeps those (souls) for which He has ordained death and sends the rest for a term appointed” (Chapter 39: verse 42),
  “…Nay, when (the soul) reaches to the collar bone (i.e. up to the throat in its exit), (26) And it will be said: “Who can cure him (and save him from death)?” (27) And he (the dying person) will conclude that it was (the time) of parting (death) (28)” (Chapter 75: verses 26-28) (The Holy Quran 2012).

The fourth argument implied by transplant advocates is reinterpreting the moral code in Abrahamic faiths to satisfy societal needs for transplantable organs. Islam shares the same fundamental rule set forth in Judaism and Christianity “Thou shalt not kill.” Islamic faith permits actions that can promote the general welfare of society or public interest (*maslaha*). The argument of *maslaha* has been commonly used to endorse the permissibility of procuring organs for transplantation (Albar 1996). Albar (1996) defines the term *maslaha* “which simply means taking care of public interest provided it does not clash with a clear text of the Quran or Sunna”. If the *maslaha* transgresses the rules of the Quran or the Sunnah, then should procuring organs be permitted to continue to satisfy the societal needs in organ transplantation? The prevention of intrinsic evil takes precedence over the promotion of good in society (Al-Allaf 2003; Padela 2007; Rady et al. 2009). Therefore, the argument of *maslaha* should not apply to actions that may be carried out because of perceived societal benefits and in good intentions but can also involve evil actions. For example, the evil resulting from ending a person’s life prematurely to recover transplantable organs should not override the good intention of saving another life. The condemnation of killing overrides the commendation on saving a life in this Quranic verse:

…if anyone killed a person not in retaliation of murder, or (and) to spread mischief in the land - it would be as if he killed all mankind, and if anyone saved a life, it would be as if he saved the life of all mankind. (Chapter 5: verse 32) (The Holy Quran 2012)

Furthermore, the divine moral code does not consider extracting organs a just cause to end someone’s life: “And do not kill anyone which Allah [God] has forbidden, except for a just cause” (Chapter 17: verse 33) (The Holy Quran 2012).

Albar (2012) has described the historical timeline of Islamic Fatwas issued on organ transplantation in Muslim communities. Although most of the Fatwas permit organ donation and transplantation, “[t]he subject of the brain death was not addressed in any of these Fatwas” (Albar 2012). On the issue of equating brain death with human death:

The Islamic League Conference of Jurists held in Makkah Al Mukaramah (December 1987), which passed Decree No. 2 (10^th^ session), did not equate cardiac death with brain death. Although it did not recognize brain death as death, it did sanction all the previous Fatwas on organ transplantation. (Albar 2012)

The Makkah Decree (1987) has rejected the earlier Resolution of the Council of Islamic Jurisprudence on Resuscitation Apparatus (1986) that equated brain death with human death. Nevertheless, the transplantation practice has largely ignored the Makkah Decree (1987) and expanded organ procurement from brain-dead patients in Muslim communities (Albar 2012).

## Islam and End-of-Life Care

In Islam, the two principles guiding end-of-life care are: (1) the sanctity of human life and (2) the dignity of the dying process and the deceased body (Athar 2011). The dignity and respect of the living and deceased human body stems from the honor that God has bestowed on man; his creation by breathing into him the soul and exalting him above all other creatures as the vice-regent on earth:

And indeed We have honoured the Children of Adam, and We have carried them on land and sea, and have provided them with At-Taiyyibat (lawful good things), and have preferred them above many of those whom We have created with a marked preferment. (Chapter 17: verse 70) (The Holy Quran 2012)

We have a moral obligation to not prolong or hasten the dying process of patients suffering from catastrophic brain injury from which, despite all appropriate medical care, they cannot recover. The Quran describes that the dying process is preordained and should not be deliberately delayed or hastened:

“And no person can ever die except by Allah’s [God] Leave and at an appointed term” (Chapter 3: verse 145),

“….but He postpones them for an appointed term and when their term comes, neither can they delay nor can they advance it an hour (or a moment)” (Chapter 16: verse 61) (The Holy Quran 2012).

However, caution must be exercised when making the clinical diagnosis of brain death. Allowing sufficient time to demonstrate the irreversible nature of the neurological injury will not have a measurable negative effect on the cost of providing medical care but will result in a better quality of care for those affected by a devastating neurological injury. Patients who have been determined brain dead are still living humans who are dying from a catastrophic irreversible injury to the brain and should not be treated as cadavers. They should be provided with appropriate end-of-life care and palliation similar to patients dying from other fatal illnesses (Schultz et al. 2012). Neurologically incapacitated patients should be entitled to the respect and dignity of a good death according to Islamic tradition (Sarhill et al. 2001; Schultz et al. 2012; Tayeb et al. 2010).

Tayeb et al. (2010) have described three important domains in the end-of-life care of dying Muslim patients: (1) religious faith; (2) self-esteem and body image; and (3) well-being of surviving family members. The first domain of religious preferences at the time of dying includes: the presence of someone to prompt the dying person to say the “*Shahadah* (bearing witness that there is no true God but Allah and Muhammad is verily His Servant and His Messenger) as a final statement of faith”; the presence of someone at bedside to recite chapters of the Holy Quran during the dying process; and positioning the dying patient to face the Kaaba at the Holy Mosque in Mecca (Tayeb et al. 2010). The second domain on self-esteem and image is preserving the bodily dignity in death by avoiding postmortem distortion of the physical body (e.g., autopsy or organ procurement), maintaining cleanliness of the body and clothing from bodily fluids (e.g., urine, stool, vomit), and ensuring normal appearance of the body after death (e.g., removing external medical devices, catheters and indwelling tubes) (Tayeb et al. 2010). The third domain is the well-being of surviving relatives, whereby the dying person feels secure that surviving family member will not be burdened with psychosocial and/or financial difficulties after one’s death (Tayeb et al. 2010).

## Alternative Solutions to Organ Transplantation

How could we address the incidence of end-stage organ (e.g., kidney, liver, heart, and lung) disease differently in society? Public health education and interventions can effectively avert the rapid rise in the incidence of end-stage organ disease through primary and secondary preventions. Primary and secondary preventative interventions can drastically decrease the incidence of end-stage heart, liver, and lung diseases and the future demand for organ transplantation. Certain lifestyles can increase the risk for future need of an organ transplant and are modifiable, such as the cessation of smoking, the abstinence from alcohol, drug addiction, and recreational substances, and the prevention of obesity. Programs that target better management of treatable chronic diseases (e.g., hypertension and diabetes mellitus) are more cost effective to society than is expanding transplantation programs in order to deal with the devastating outcome from neglected medical care. We think that the supply of organs can never satisfy the transplantation demand unless society prioritizes the expansion of preventative healthcare programs instead of organ transplantation. Organ transplant should medically be considered palliative rather than curative treatment of end-stage organ disease for at least two reasons. First, kidney transplant, for example, is not uniquely life-saving since hemodialysis is an alternative treatment option to preserve life. Kidney transplant is generally credited for improved the quality of life of recipients as compared to dialysis-dependent patients. Second, the chronic rejection of the transplanted organ secondary to the recipient immune system and the long-term toxic effects of immunosuppressive drugs inevitably result in deteriorating function of the donated organ (Nankivell and Kuypers 2011). Immunosuppressive drugs can also cause serious medical complications leading to premature death (Jardine et al. 2011). Immunosuppressive drugs can exacerbate chronic conditions such as diabetes mellitus, hypertension, hyperlipidemia, and atherosclerosis with premature incidence of cardiovascular and cerebrovascular diseases in transplant recipients (Jardine et al. 2011). Finally, suppression of the immune system increases the risk of life-threatening new malignancies and infections.

With regard to the retrieval of transplantable organs, society must have a transparent debate on the sociocultural consequences of expanding an organ-donation practice that is currently grounded in a scientifically flawed criterion of death. Furthermore, a first-person explicit consent or presumed consent system for organ donation is not legally or ethically acceptable in Muslim communities unless society has accepted and agreed to legalize physician-assisted death in organ donation practice (e.g., a person can legally consent to death by organ donation) (Verheijde and Rady 2011). This type of legislation will contravene the fundamental values and laws of Islamic faith.

## Conclusions

In conclusion, brain death, as synonymous with human death, has been deeply rooted in Western medical practice as a prerequisite for advancing human organ transplantation. The utilitarian definition of death in organ donation into two types, neurological (heart-beating donation) and circulatory (non-heart-beating donation), diverges from the singularity of both the biological phenomenon as well as the theological definition of death. The societal implications of violating basic human rights of the vulnerable dying persons and transgressing their religious beliefs are catastrophic. Therefore, it is imperative to reevaluate the two different types and criteria of death introduced by the Resolution (Fatwa) of the Council of Islamic Jurisprudence on Resuscitation Apparatus in 1986. Fatwas permitting end-of-life organ donations were based on currently outdated knowledge and understanding of the phenomenon of human death. The definition of death in Islam should reaffirm the singularity of human death as revealed in the Quran 14 centuries ago.
